# Monitoring and Identifying Emerging e-Cigarette Brands and Flavors on Twitter: Observational Study

**DOI:** 10.2196/42241

**Published:** 2022-12-05

**Authors:** Qihang Tang, Runtao Zhou, Zidian Xie, Dongmei Li

**Affiliations:** 1 Goergen Institute for Data Science University of Rochester Rochester, NY United States; 2 Department of Clinical & Translational Research University of Rochester Medical Center Rochester, NY United States

**Keywords:** e-cigarettes, brand, flavor, Twitter

## Abstract

**Background:**

Flavored electronic cigarettes (e-cigarettes) have become very popular in recent years. e-Cigarette users like to share their e-cigarette products and e-cigarette use (vaping) experiences on social media. e-Cigarette marketing and promotions are also prevalent online.

**Objective:**

This study aims to develop a method to identify new e-cigarette brands and flavors mentioned on Twitter and to monitor e-cigarette brands and flavors mentioned on Twitter from May 2021 to December 2021.

**Methods:**

We collected 1.9 million tweets related to e-cigarettes between May 3, 2021, and December 31, 2021, by using the Twitter streaming application programming interface. Commercial and noncommercial tweets were characterized based on promotion-related keywords. We developed a depletion method to identify new e-cigarette brands by removing the keywords that already existed in the reference data set (Twitter data related to e-cigarettes from May 3, 2021, to August 31, 2021) or our previously identified brand list from the keywords in the target data set (e-cigarette–related Twitter data from September 1, 2021, to December 31, 2021), followed by a manual Google search to identify new e-cigarette brands. To identify new e-cigarette flavors, we constructed a flavor keyword list based on our previously collected e-cigarette flavor names, which were used to identify potential tweet segments that contain at least one of the e-cigarette flavor keywords. Tweets or tweet segments with flavor keywords but not any known flavor names were marked as potential new flavor candidates, which were further verified by a web-based search. The longitudinal trends in the number of tweets mentioning e-cigarette brands and flavors were examined in both commercial and noncommercial tweets.

**Results:**

Through our developed methods, we identified 34 new e-cigarette brands and 97 new e-cigarette flavors from commercial tweets as well as 56 new e-cigarette brands and 164 new e-cigarette flavors from noncommercial tweets. The longitudinal trend of the e-cigarette brands showed that JUUL was the most popular e-cigarette brand mentioned on Twitter; however, there was a decreasing trend in the mention of JUUL over time on Twitter. Menthol flavor was the most popular e-cigarette flavor mentioned in the commercial tweets, whereas mango flavor was the most popular e-cigarette flavor mentioned in the noncommercial tweets during our study period.

**Conclusions:**

Our proposed methods can successfully identify new e-cigarette brands and flavors mentioned on Twitter. Twitter data can be used for monitoring the dynamic changes in the popularity of e-cigarette brands and flavors.

## Introduction

Electronic cigarettes (e-cigarettes) or vapes are electronic devices that heat and aerosolize liquids containing propylene glycol, vegetable glycerin, flavorings, and in most cases, nicotine [1]. Although e-cigarettes have been marketed as safer and healthier alternatives to help adult smokers quit smoking, e-cigarettes have become very popular among teenagers who have never smoked [2]. Based on data from the Centers for Disease Control and Prevention, 11.3% of high school students and 2.8% of middle school students in the United States reported using e-cigarettes in 2021. More than a quarter of the current e-cigarette users in high school reported daily e-cigarette use [3]. Although previous studies have shown that e-cigarettes might be less harmful than smoking due to the low level of toxic chemicals released during their usage, they can still harm adolescents’ brains [4,5]. The nicotine usually contained in e-cigarettes is highly addictive and can affect the development of the parts of the brain that control attention, learning, mood, and impulse control, which can continue affecting up to the early age of 25 years [6,7]. In addition, nicotine use may increase the risk of adolescents’ addictions to other drugs in the future [7]. A previous study has found that for people with chronic obstructive pulmonary disease or asthma [8], using e-cigarettes regularly can potentially accelerate their disease progression.

To protect public health in the general population, especially in youth, different tobacco policies have been announced to intervene and regulate e-cigarette products to prevent excessive e-cigarette use among teenagers. On December 20, 2019, a federal law was enacted to raise the federal minimum legal sales age from 18 years to 21 years for all tobacco products, including e-cigarettes, across the United States [9]. This policy makes it harder for teenagers to legally access e-cigarettes. One study has reported that adolescents and young adults described appealing flavors as their leading reason to use e-cigarette products [10]. Thus, several policies have been implemented to regulate e-cigarette flavors. On January 2, 2020, the Food and Drug Administration (FDA) announced the flavor enforcement policy, which was later implemented on February 6, 2020, to ban the sale of all unauthorized flavored cartridge-based e-cigarette products, except tobacco and menthol-flavored products [11]. Following the FDA flavor enforcement policy on May 18, 2020, the New York state banned all e-cigarette flavors, except the tobacco flavor [12].

Although many tobacco regulation policies are in place to reduce the vaping epidemic, especially among youth in recent years, the e-cigarette industry is trying to find a loophole to increase the sales of e-cigarette products through aggressive marketing on social media. Therefore, it is of the utmost importance to monitor the real-time dynamic changes in e-cigarette brands and flavors available in the market. With timely information on the prevalence of popular e-cigarette brands and flavors, regulatory authorities and policy makers could take immediate actions to help reduce the vaping epidemic, such as by issuing warning letters to unauthorized emerging e-cigarette products that are becoming popular. Twitter is one of the largest and most influential social media platforms, which had around 48.35 million active users in the United States in 2019 [13]. More importantly, Twitter allows e-cigarette users to share their experiences with e-cigarette products and discuss the government’s e-cigarette–related policies and regulations [14]. In addition, vaping products have been actively promoted online, especially on social media by the vaping industry. With aggressive social media marketing, JUUL has successfully grown from a little-known brand in 2015 to the largest e-cigarette retailer in 2017, and similar social media marketing strategies have been used by many other e-cigarette brands [15]. It was estimated that in 2021, about 7 in 10 middle school and high school students were exposed to e-cigarette advertising [16]. Previous studies have leveraged social media data to study e-cigarette–related topics. For example, Sun et al [17] demonstrated that Twitter data could be used to study the promotions of various disposable e-cigarette flavors and related discussion topics. Lu et al [18] showed the feasibility of utilizing Reddit data to monitor the popularity of e-cigarette flavors based on a list of known e-cigarette flavors. A named-entity recognition model was proposed to identify e-cigarette brands and flavors from Instagram posts [19]. However, there is no effective approach to identify new e-cigarette brands or flavors in the market. With the dynamic changes in e-cigarette products, it is very important to identify and monitor new e-cigarette brands or flavors that are emerging and becoming popular in the market. The newly identified e-cigarette brands and flavors along with the popularity of different brands and flavors can be a useful asset for the government to make informed policy to reduce the vaping epidemic.

In this study, using Twitter data, we monitored the longitudinal trend of mention of e-cigarette brands and flavors in commercial and noncommercial tweets. More importantly, we identified new e-cigarette brands and flavors mentioned on Twitter. Therefore, our findings provide important insights into the dynamic e-cigarette landscape with new e-cigarette brands and flavors, which will help policymakers or tobacco regulators to enforce better regulations for public health protection.

## Methods

### Data Collection and Preprocessing

Through the Twitter streaming application programming interface, we collected e-cigarette–related Twitter posts (tweets) between May 3, 2021, and December 31, 2021, by using a set of e-cigarette–related keywords such as e-cigarette, e-cig, vaping, and e-liquid [18,20]. Duplicated tweets were removed from the data set. There were 1,932,707 e-cigarette–related tweets collected in total. By filtering with different keywords (such as e-cig, vaping, supply, and deal) from the names of the users who posted the tweets and promotion-related keywords (such as discount and free shipping) from the contents of the tweets, the tweets were characterized as commercial tweets (tweets that are from retailers or contain promotion information). The remaining tweets were characterized as noncommercial tweets (general discussions of e-cigarette–related topics). During this process, the language of the content of the tweets was evaluated, and only English tweets were kept. During the period between May 3, 2021, and December 31, 2021, there were 285,170 commercial tweets and 1,472,928 noncommercial tweets. From September 1, 2021, to December 31, 2021, there were 139,199 commercial tweets and 728,284 noncommercial tweets. Among all the tweets between May 3, 2021, and December 31, 2021, 890,209 tweets were retweets (tweets that were originally composed by other users and were reposted). These retweets were used for new brand and flavor identification but not for monitoring the popularity of the recorded brands and flavors.

### Ethics Approval

This study has been reviewed and approved by the Office for Human Subject Protection Research Subjects Review Board at the University of Rochester (study ID: STUDY00006570).

### New e-Cigarette Brand Identification

We collected and constructed an identified list of the available e-cigarette brands and flavors by searching web-based stores, which contained 129 e-liquid brands and 1198 e-liquid flavors [18]. To identify possible new e-cigarette brands mentioned on Twitter, we developed a depletion approach. For this, e-cigarette Twitter data from May 3, 2021, to August 31, 2021, were used as the reference data set and Twitter data from September 1, 2021, to December 31, 2021, were used as the target data set to identify new e-cigarette brands. As shown in Figure S1 in Multimedia Appendix 1, a reference single-word list was constructed using the reference data set (e-cigarette Twitter data from May 3, 2021, to August 31, 2021) by tokenizing the words and recording the frequency of appearance of each of the unique word tokens. Two target single-word lists (commercial and noncommercial single-word lists) were constructed using the same approach from the target Twitter data set (Twitter data from September 1, 2021, to December 31, 2021). By removing the word tokens of the target single-word lists that appeared in the reference single-word list or the identified brand list, 2 depleted lists (commercial and noncommercial lists) were generated. The 2 depleted lists were then manually examined through Google search to check if each word token referred to an e-cigarette brand. As there were more than 50,000 keywords that were mentioned less than 10 times, only keywords mentioned at least 10 times were manually checked considering the time constraints. We considered the word token as the mentioning of a brand if the word was either a brand name or a product name within the brand. If the word token was only a part of the brand name and the word token was referring to the brand, we also considered the word as the mention of the brand. To verify newly identified brands, we conducted a Google search to ensure that these brands have been listed in e-cigarette–related websites or web-based stores.

### New e-Cigarette Flavor Identification

As shown in Figure S2 in Multimedia Appendix 1, new flavor identification was done differently from brand identification, and the whole collected data set from May 3, 2021, to December 31, 2021, was used to identify new e-cigarette flavors. The type of tweets, either commercial or noncommercial, was tracked during the identification. All tweets containing the key phrase “new flavor” were directly saved as new flavor candidates. A flavor keyword list was then constructed by using unique single words that appeared in the identified list of our previously collected e-cigarette flavors [18]. Tweets were split into different segments by punctuation and the keyword “and” to deal with the possibility of multiple flavors mentioned in the same tweet. As flavor names were likely to contain certain common flavor keywords, segments that contained one or more flavor keywords were more likely to contain e-cigarette flavors. By removing segments that contained flavors that were already existing in our previous identified flavor list, the remaining tweet segments became the candidates for potential new flavors. We conducted a similar Google search to make sure that these newly identified flavors are existing e-cigarette flavors. The newly identified e-cigarette brands and flavors determined by the methods mentioned above were added to our e-cigarette brand or flavor list.

### e-Cigarette Brand and Flavor Monitoring

To determine if each e-cigarette–related tweet contains an e-cigarette brand or flavor, each tweet was split into segments based on punctuations. Then, we searched each e-cigarette brand name or flavor name in our constructed brand or flavor database that contained brands and flavors from both the original database and the newly identified ones from the target Twitter data set in each segment based on exact match (case insensitivity). We then calculated the number of tweets mentioning each e-cigarette brand and flavor in the commercial and noncommercial tweets each month.

## Results

### Identification of New e-Cigarette Brands on Twitter

**Figure 1:**
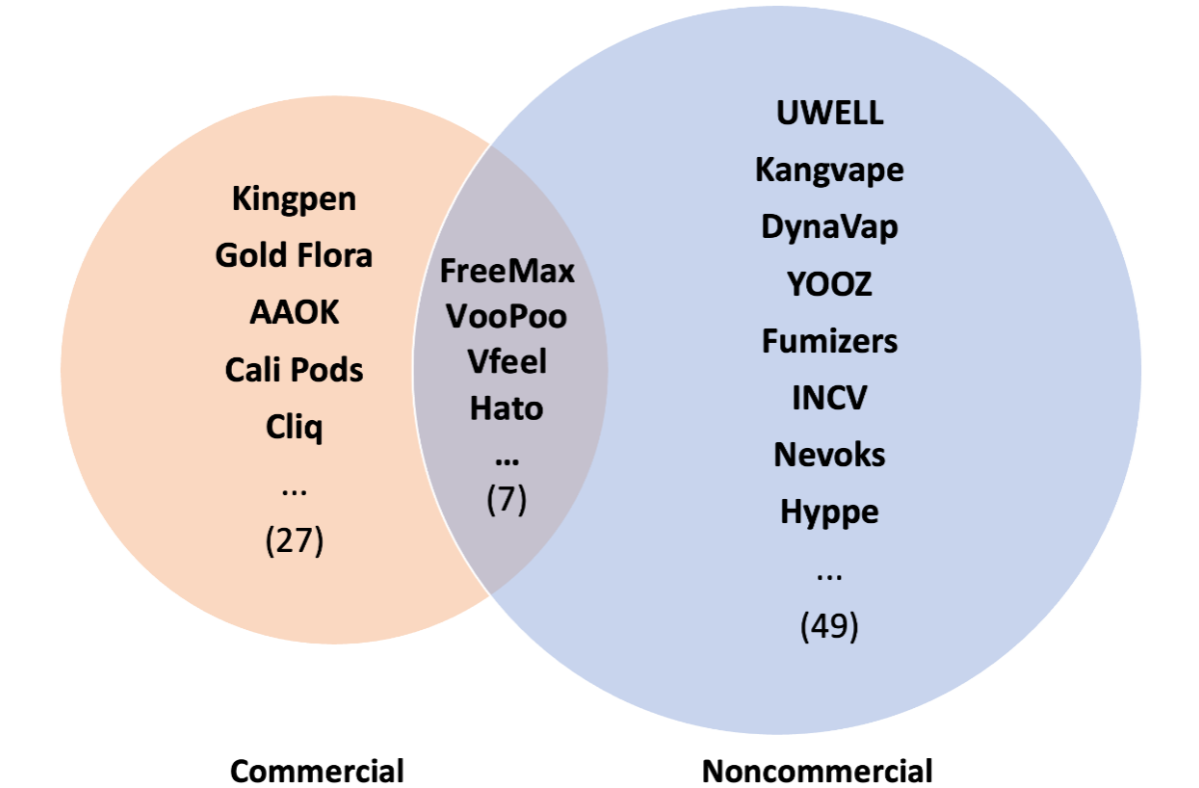
New electronic cigarette brands mentioned on Twitter.

Using the depletion methods, we identified 27,012 candidate words from commercial tweets and 62,117 candidate words from noncommercial tweets between September 1, 2021, and December 31, 2021, which were not present in tweets between May 3, 2021, and August 31, 2021. Among these candidate words, 831 commercial candidate words and 1966 noncommercial candidate words appeared at least 10 times. After manually searching those words with frequencies over 10 times online, we identified 34 new e-cigarette brands (such as Kingpen and Gold Flora) from the commercial tweets and 56 new e-cigarette brands (such as Uwell, Kangvape, and DynaVap) from the noncommercial tweets. Among the newly identified brands, 7 e-cigarette brands (ie, FreeMax, VooPoo, Vfeel, Hato, Innokin, Vaporesso, and Vaptex) were identified from both the commercial and the noncommercial tweets (Figure 1 and Table S1 in Multimedia Appendix 1).

### Longitudinal Monitoring of e-Cigarette Brands Mentioned on Twitter

**Figure 2:**
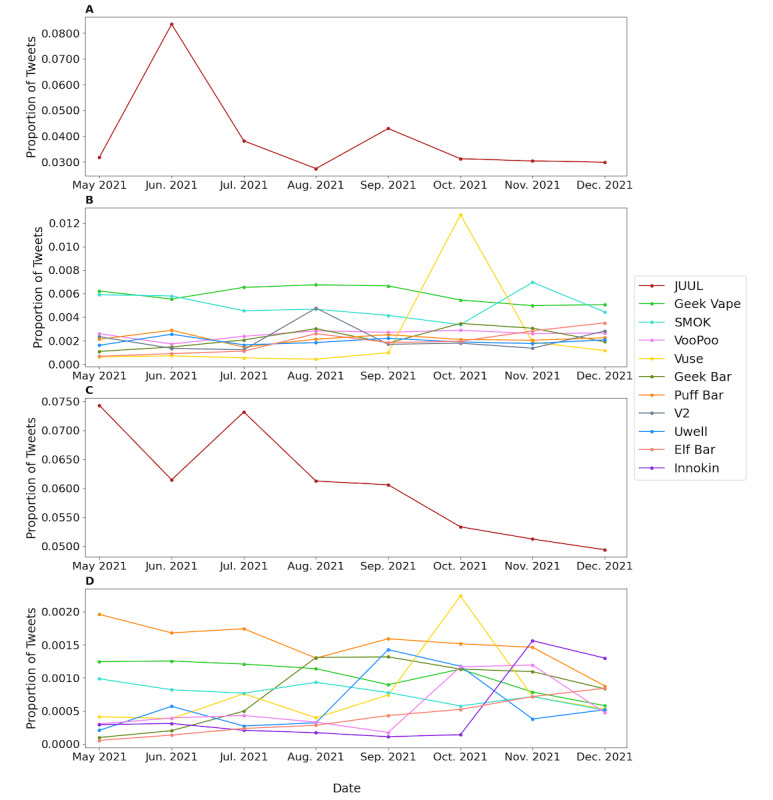
The longitudinal trends of electronic cigarette brands mentioned on Twitter. A: JUUL in commercial tweets. B: The other top 9 electronic cigarette brands in commercial tweets. C: JUUL in noncommercial tweets. D: The other top 9 electronic cigarette brands in noncommercial tweets.

To monitor the dynamic changes of each e-cigarette brand mentioned on Twitter, we calculated the proportion of tweets mentioning each e-cigarette brand over time in both commercial and noncommercial tweets. Due to the significant difference between the popularity of JUUL and other e-cigarette brands, we put JUUL in separate graphs to better show the trends of JUUL and other brands (Figure 2). As shown in Figure 2A and Figure 2C, the proportion of e-cigarette–related tweets mentioning JUUL was the highest among all e-cigarette brands over time in both commercial and noncommercial tweets. In the commercial tweets, the monthly mentions of JUUL were steady between 3% and 5%, with an exceptionally high proportion of over 8% in June 2021 (Figure 2A). In the noncommercial tweets, the monthly mentions of JUUL showed a decreasing trend (Figure 2C). In both commercial and noncommercial tweets (Figure 2B and 2D), the monthly mentions of Vuse were roughly steady, with an exceptional peak around 1.27% (commercial) and 0.2% (noncommercial) in October 2021. Although it was not a top mentioned brand in commercial tweet, Innokin seemed to be a popular brand mentioned by e-cigarette users in noncommercial tweets, which was obviously popular in November and December 2021.

### Identification of New e-Cigarette Flavors on Twitter

**Figure 3:**
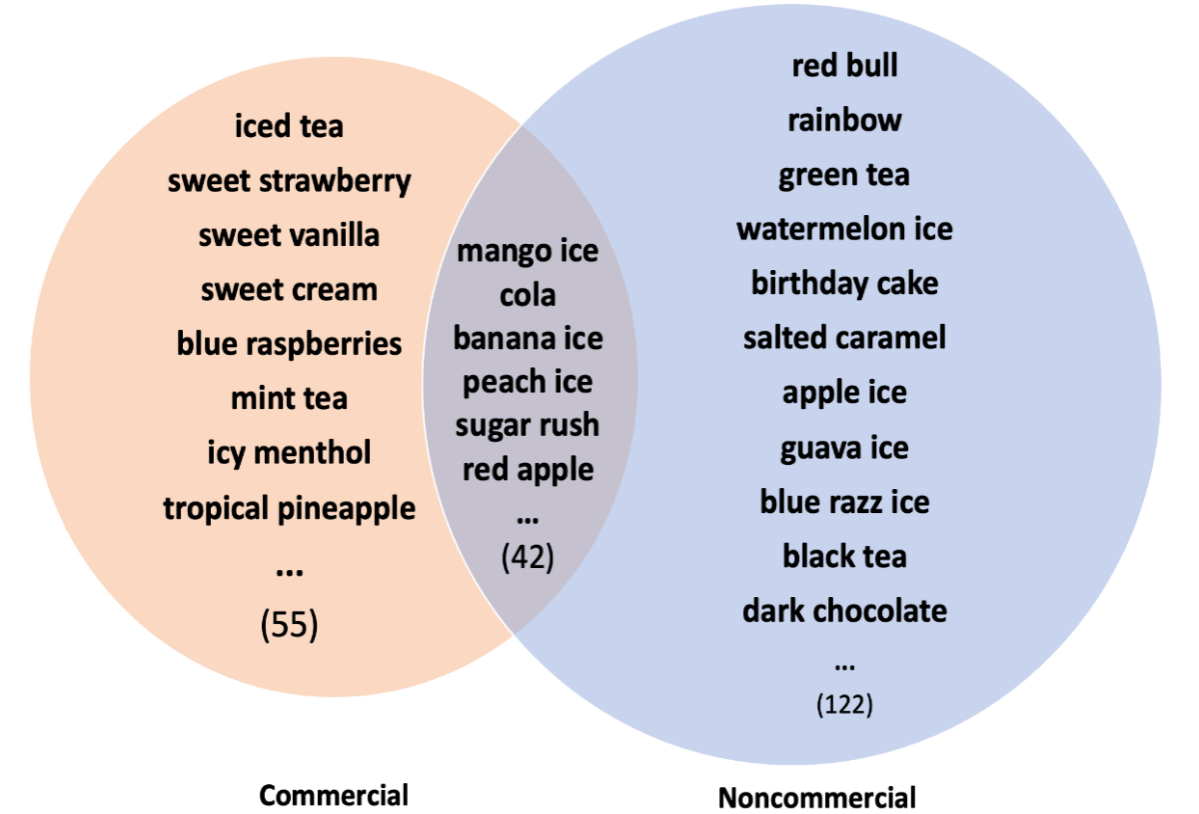
New electronic cigarette flavors mentioned on Twitter.

With the quick evolvement in the e-cigarette market due to different flavor regulations and marketing needs, it is important to identify new e-cigarette flavors appearing in the market. By comparing to our previously collected e-cigarette flavor lists, from our e-cigarette Twitter data set (May 3 to December 31, 2021), we identified 1145 flavor candidates from commercial tweets and 3736 candidates from noncommercial tweets. By manually verifying all the flavor candidates, we identified 97 new e-cigarette flavors (such as iced tea, sweet strawberry, and sweet vanilla) from commercial tweets and 164 new flavors (such as red bull, rainbow, and green tea) from noncommercial tweets (Figure 3 and Table S2 in Multimedia Appendix 1). Among them, 42 new e-cigarette flavors (such as mango ice, cola, and banana ice) were identified from both commercial and noncommercial tweets (Figure 3 and Table S3 in Multimedia Appendix 1).

### Longitudinal Monitoring of e-Cigarette Flavors Mentioned on Twitter

**Figure 4:**
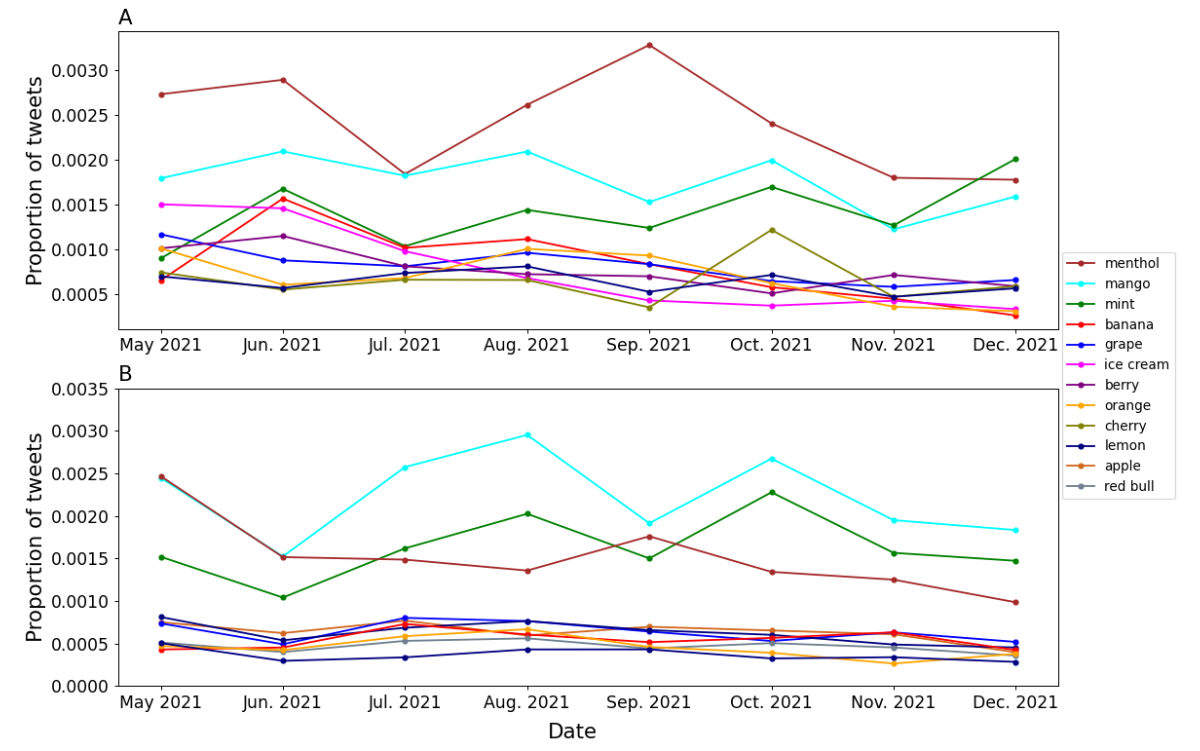
The longitudinal trend of electronic cigarette flavors mentioned on Twitter. A: Commercial tweets. B: Noncommercial tweets.

With different flavor regulations on e-cigarettes and the dynamic changes in the e-cigarette market, the mentions (or even the use) of different e-cigarette flavors on Twitter might be evolving. As shown in Figure 4, in both commercial and noncommercial tweets, mango, menthol, and mint were the most popular e-cigarette flavors. The trends in e-cigarette flavors were represented as the proportions of tweets mentioning each flavor in the total number of e-cigarette–related tweets within each month. The top 10 most popular e-cigarette flavors mentioned in both commercial and noncommercial tweets generally had the same popularity with some variation from May 3, 2021, to December 31, 2021. In the commercial tweets, the menthol flavor was the most popular e-cigarette flavor, followed by mango and mint flavors (Figure 4A). In the noncommercial tweets, the mango flavor was the most popular e-cigarette flavor mentioned, followed by menthol and mint flavors (Figure 4B).

## Discussion

### Principal Findings

With the rapid change in the e-cigarette landscape, it is important to monitor not only the existing but also the newly emerging e-cigarette brands and flavors. In this study, using Twitter data, we developed a depletion method to identify newly emerging e-cigarette brands and a keyword identification method to identify newly emerging e-cigarette flavors based on Twitter data. Using the developed methods, we identified 76 new e-cigarette brands that were not included in our previously collected e-cigarette brands [18]. In addition, we identified 177 new e-cigarette flavors that were not present in our previously collected e-cigarette flavors. Furthermore, we monitored the trends of e-cigarette brands and flavors mentioned in both commercial and noncommercial tweets from May 3, 2021, to December 31, 2021, separately. In both commercial and noncommercial tweets, the top mentioned e-cigarette brands and flavors were similar. JUUL was the most prevalent brand, and mango, menthol, and mint were the most popular flavors mentioned in both commercial tweets and noncommercial tweets. With the dynamic changes in the e-cigarette brands in the market, it is necessary to identify not only the existing but also the newly emerging e-cigarette brands and flavors to monitor the changes in the e-cigarette market. However, as the brand names can be any word and phrase without any obvious common pattern, it is very difficult to identify potential brand names directly from tweets. As a result, instead of directly identifying potential brand names, our method tries to exclude all the word tokens, which were neither related to any e-cigarette brand nor related to e-cigarette brands that are already recorded, during the depletion process. All the word tokens left may therefore be related with e-cigarette brands that are not in our recorded list. As the list of the general English words checked and the list of the known e-cigarette brands are being constantly updated and enlarged, our method will become more accurate and thus will require less human labor. Different from e-cigarette brand names, e-cigarette flavor names are more likely to contain certain keywords, especially when new flavors are more likely to be the mixture of single flavors that are mostly likely included in our previously collected flavor list.

### Comparison With Prior Work

Previous studies have taken different approaches to monitor e-cigarette–related activities and entities on social media platforms. Several studies have developed named-entity recognition models to identify emerging brands and flavors on Instagram. For example, Chew et al [19] were able to identify brands with an F1-score of 0.815 and flavors with an F1-score of 0.828 with fine-tuned distilled bidirectional encoder representations from transformers network. Kavuluru and Sabbir [21], on the other hand, developed a proponent classification model to identify e-cigarette proponents (users who advocate e-cigarette publicly with their account) on Twitter. Another study developed a cluster model to identify the co-occurrence of different e-cigarette–related hashtags and the geographical information where e-cigarette–related discussions occurred [22]. However, all the known prior studies took the approach to either monitor only the known e-cigarette brands and flavors or to identify the related information (proponents, hashtags, locations, etc). To the best of our knowledge, no study has tried to identify newly emerging e-cigarette brands and flavors. Our research has introduced a novel approach to identify emerging e-cigarette brands and flavors in the market. More importantly, we created a method that systematically monitors the popularity of different e-cigarette brands and flavors longitudinally. As Chew et al [19] suggested, new brands are entering the marketplace by first promoting on social media, and therefore, relying only on the traditional data source (such as Nielsen sales data) may not be enough. In this study, we developed some useful approaches to identify new e-cigarette brands and flavors mentioned on Twitter, which will help us better understand the dynamic changes in the e-cigarette market. Different from the classification methods or clustering models developed in a prior study [19], our methods do not include any machine learning approach and therefore do not have evaluation metrics (such as F1-score) that can be used to directly compare with prior work. Theoretically, our methods could also be easily adapted to identify new brands or flavors for other tobacco products (such as waterpipe and oral nicotine pouches) or other nontobacco products by using social media data. Although our methods require human involvement at the final step, human involvement is likely to significantly decrease as more Twitter data are analyzed and added into the reference Twitter data set.

The motivation for monitoring e-cigarette brands and flavors is to demonstrate the effect of government policy changes on people’s perception of e-cigarettes. One direct way to detect the people’s perception changes on e-cigarettes is by looking at the monthly popularity trends of e-cigarette brands and flavors. Many previous studies have focused on the public perceptions of government regulation on e-cigarettes. On January 2, 2020, the FDA released an e-cigarette enforcement policy, which prohibited the sale of all flavored cartridge-based e-cigarettes, except for menthol and tobacco flavors [11]. One study showed that the government’s flavor ban might have changed the public perceptions of e-cigarettes by using Twitter data [23]. Another study concentrated public reaction on New York state e-cigarette flavor ban on September 17, 2019. Both studies indicated that after the policy was announced, people tended to have a more negative attitude toward e-cigarettes on Twitter [12]. Those studies demonstrated that public reactions to different governments’ e-cigarette flavor policies can be detected using tweets. In this study, we showed that although JUUL is the most popular e-cigarette brand in terms of the number of tweets mentioned per month by a large margin, its number of mentions showed a general declining trend. By contrast, e-cigarette brands such as Elf Bar, Geek Bar, and Vuse appeared to have an increasing number of tweets mentioned per month. One reason to explain the downward trend is JUUL’s limited flavor options for their products. JUUL has been criticized by the public for directly target advertising toward youth since 2015 [24]. Studies have shown that at its peak in 2018, 31% of the JUUL users were younger than 18 years [25]. Part of JUUL’s popularity among teenagers comes from the fact that JUUL used to produce a wide variety of nontraditional-flavored e-cigarette products [24]. Nontraditional-flavored e-cigarettes are more appealing to teenagers compared to the traditional-flavored e-cigarette counterparts, as they provide a sensory perception of sweetness and coolness [26]. However, due to increasing public pressure against JUUL’s appealing flavor products, which attract adolescents, in November 2018, JUUL voluntarily restricted their sales of mango, fruit medley, and cucumber pods on its website [27]. In October 2019, JUUL lab announced that it would suspend sales of all nontobacco- and nonmenthol-based flavors in the United States [28]. In January 2020, FDA’s flavor enforcement policy on all unauthorized cartridge-based flavored e-cigarettes made it impossible for JUUL to bring back their most loved fruit-flavored products like mango JUUL pods [11,29]. Studies have shown that ever since JUUL announced an end to the sale of their flavored e-cigarette products, their relative search volume on Google dropped steadily at a pace of 8.8 relative search volume per week. However, the relative search volume of disposable e-cigarette brands like Elf Bar, Puff Bar, and Geek Bar showed an increasing trend over time [30], which roughly corresponds to the trend shown in Figure 2B. These trends demonstrated that people are gradually replacing cartridge-based e-cigarette brands like JUUL with disposable e-cigarette brands like Puff Bar, Geek Bar, and Elf Bar to circumvent the FDA’s flavor ban. Another way for e-cigarette companies to circumvent the FDA flavor regulations is to introduce the “concept flavor” (vague noncharacterizing descriptions on the packaging that do not expressly refer to real flavors) to rename their products [31]. For example, the popular disposable e-cigarette brand BIDI Stick unveiled a dozen new flavors, including Zest (formerly Jungle Juice), Arctic (formerly Mint freeze), and Solar (Berry Blast), among others. On June 23, 2022, the FDA issued market denial orders to JUUL Lab Inc for all their products currently marketed in the United States due to JUUL’s lack of sufficient evidence regarding the toxicological profile of their products to demonstrate that they meet the public health standards [32].

Although JUUL was dominant on Twitter during our study period, Vuse is a top e-cigarette brand, and its monthly trends showed an exceptionally high peak in October 2021 in contrast to the stable or slight downward trend of other brands. One potential reason for this peak is the approval announcement of the FDA in October 2021. The FDA announced on October 12, 2021, that it would allow the selling of Vuse Solo e-cigarette in the United States, making Vuse the first authorized e-cigarette brand for sale in the United States [33]. In response to this announcement, it was very likely that people were discussing Vuse and the decision of FDA in the same month. In addition, e-cigarette retailers and other commercial Twitter accounts were posting promotion tweets about Vuse, leading to the exceptionally high frequencies of mention in both commercial and noncommercial tweets. Although Twitter data may not fully reflect the e-cigarette users and retailer groups, our results demonstrated that the effect of FDA’s announcements and policies on e-cigarette brands and flavors on general users and retailers can be monitored through monitoring the existing and newly emerging e-cigarette brands and flavors on Twitter.

Our data showed that almost all the top 10 e-cigarette flavors, including menthol, grape, orange, and banana, experienced a general downward trend in terms of number of mentions in commercial tweets. This indicates that flavored e-cigarettes have been promoted lesser on Twitter in response to the FDA e-cigarette flavor regulation policies [34]. FDA has restricted the Premarket Tobacco Product Applications approval since February 2020, which greatly reduced the number of new flavored e-cigarette products entering the e-cigarette market. In total, the FDA agencies have issued 263 marketing denial orders to more than 1 million flavored e-cigarette products [35]. However, despite the FDA e-cigarette flavor regulation enforced in February 2020, there are still flavored e-cigarette product promotions on Twitter. In an effort to circumvent the FDA e-cigarette flavor regulation, 6 big manufacturers of 98 different brands, including Buff Bar and Geek Bar, claimed that their products contain synthetic nicotine [36]. FDA did not enforce any flavor regulation policies on e-cigarette products containing synthetic nicotine. Thus, it is reasonable for vaping companies or stores to promote their flavored e-cigarette products containing synthetic nicotine on Twitter. Another way to avoid FDA e-cigarette regulations is to sell disposable e-cigarettes. Since the FDA flavor regulations did not include disposable e-cigarette devices [11], vaping companies or vaping stores can still promote their flavored disposable e-cigarette on Twitter. The Twitter data used in this study includes disposable e-cigarettes. Thus, e-cigarette flavors mentioned in commercial tweets may come from disposable e-cigarette product promotions.

### Limitations

Although the Twitter data analyzed in this study have a large sample size, the trends in e-cigarette brands and flavors on Twitter may not reflect the trends in the real world, as Twitter users cannot represent all the population around the globe. The e-cigarette brands and flavors that have never been mentioned on Twitter will not be captured. For both methods identifying new e-cigarette brands and flavors proposed in this study, there are several limitations. First, both methods require sufficient prior knowledge. New e-cigarette brand identification requires enough tweets being labeled in order to be used in the depletion method to reduce the noise in the resulting candidate list. In this study, the first half of the e-cigarette–related Twitter data set is assumed to be the reference data and has not been manually checked. Unidentified e-cigarette brands in the reference data set may not be captured if these brands are never mentioned in the target data set. As many general English words are removed through depletion, names of e-cigarette brands that are formed by general English words may not be captured. Flavor identification requires enough known flavors to capture enough flavor keywords. For any new flavor wherein the name does not have any overlap with the names in our previously recorded list, if they are mentioned in tweets without the keyword “new flavor,” they will not be captured. Second, both methods generate long candidate lists and require a large amount of human labor, which need to be further improved with the help of deep learning techniques. Although there is an increasing popularity of “concept flavor” for e-cigarettes to avoid the flavor regulatory policies, we do not capture these flavors as they do not fall into the standard flavor names, which need to be further examined in future studies.

### Conclusion

In this paper, we proposed a new method to identify new e-cigarette brands and flavors. This study is one of the first attempts to use Twitter data to discover new e-cigarette brands and flavors. We have successfully identified 62 new e-cigarette brands and 305 new e-cigarette flavors mentioned on Twitter. More importantly, we were able to monitor the dynamic changes in e-cigarette brands and flavors mentioned on Twitter over time (both commercial and noncommercial). Our results demonstrate that Twitter data can be used to discover new e-cigarette brands and flavors as well as provide real-time surveillance on e-cigarette brands and flavors in the market.

## Appendix

Multimedia Appendix 1 Supplementary tables and figures.

## Abbreviations

e-cigarette: electronic cigarette
FDA: Food and Drug Administration

## Data Availability

The data sets generated during or analyzed in this study are available from the corresponding author on reasonable request.
