# Supplementary material for: Monitoring and Identifying Emerging e-Cigarette Brands and Flavors on Twitter: Observational Study
Source: JMIR Form Res. 2022 Dec 5;6(12):e42241. doi: 10.2196/42241 (PMC9764155; doi:10.2196/42241)
Supplement: Multimedia Appendix 1 [file formative_v6i12e42241_app1.docx]

**Table S1.** Newly identified e-cigarette brands.

| **Commercial** | | **Non-commercial** | |
| --- | --- | --- | --- |
| **Brand Name** | **Frequency** | **Brand Name** | **Frequency** |
| FreeMax | 97 | Innokin | 242 |
| VooPoo | 90 | Uwell | 238 |
| Kingpen | 72 | Kangvape | 222 |
| Gold Flora | 61 | DynaVap | 132 |
| AAOK | 38 | YOOZ | 128 |
| Vfeel | 34 | Fumizers | 105 |
| Cali Pods | 31 | INVC | 102 |
| Cliq | 30 | Nevoks | 96 |
| iJOY | 28 | Hyppe | 84 |
| Supherb | 23 | Omari-O | 75 |
| Hippo | 23 | Wismec | 74 |
| Tenx | 20 | Nectar Collector | 55 |
| Hato | 19 | FreeMax | 55 |
| Terpen | 15 | Hato | 44 |
| Flexvape | 15 | VapX | 42 |
| B+MOR / BMOR | 14 | Storz & Bickel | 41 |
| Innokin | 14 | Vfeel | 38 |
| FANK | 13 | Joymy | 38 |
| Vaporesso | 13 | VooPoo | 38 |
| Cabissi | 12 | Select | 36 |
| Mlife | 12 | Kumiho | 35 |
| Vaptex | 11 | Whisl | 35 |
| Hale | 11 | Joyetech | 34 |
| IFRIT | 10 | Trailblazer | 30 |
| Hyppe | 10 | Ikhal | 29 |
| Lula Vape | 10 | Vapelustion | 27 |
| SHFT | 9 | Little Dipper | 25 |
| Biasing | 9 | MedPharm | 24 |
| Zuk Vape | 9 | MaxCore | 23 |
| Wuuz | 9 | MGO | 23 |
| Paladin | 9 | CloudV | 23 |
| Mig Vapor | 8 | Greenlightvapes | 21 |
| Cevo | 8 | midose | 21 |
| VYKO | 7 | Advken | 21 |
|  |  | Freeton | 21 |
|  |  | Vaporesso | 20 |
|  |  | Vape Canyon | 18 |
|  |  | NOMS | 18 |
|  |  | JuJu Royal | 18 |
|  |  | Coee | 18 |
|  |  | BeatNic Vapor | 17 |
|  |  | Vaptex | 17 |
|  |  | Mods | 16 |
|  |  | MPX / Melting Point Extracts | 14 |
|  |  | Amigo Itsuwa | 13 |
|  |  | Strangers Mods | 13 |
|  |  | Hookah | 13 |
|  |  | Blitz | 12 |
|  |  | VapeFly | 11 |
|  |  | ILEVA | 11 |
|  |  | CCELL | 10 |
|  |  | Pure Extracts | 10 |
|  |  | Yocan | 10 |
|  |  | Fizzy | 10 |
|  |  | Cali Greens | 10 |
|  |  | Eleaf | 10 |

*The frequency in this graph represents the frequency of the candidate words that helps us identify the corresponding brands. The precise frequencies of each brand are shown in the monthly trend graphs.

**Table S2**. Newly identified e-cigarette flavors on Twitter.

| **Non-commercial** | | **Commercial** | |
| --- | --- | --- | --- |
| **Flavor** | **Frequency** | **Flavor** | **Frequency** |
| red bull | 643 | ice coffee | 87 |
| rainbow | 266 | iced tea | 35 |
| mountain dew | 128 | mint tea | 18 |
| green tea | 93 | sweet strawberry | 18 |
| birthday cake | 50 | milk chocolate | 8 |
| watermelon ice | 49 | juicy peach | 7 |
| cookie dough | 47 | blue raspberries | 7 |
| waffle | 42 | chocolate cake | 7 |
| milk tea | 26 | strawberry guava | 5 |
| apple ice | 23 | coconut milk | 5 |
| black tea | 18 | sour gummy | 5 |
| guava ice | 18 | juicy grapes | 5 |
| glazed donut | 15 | sweet honey | 5 |
| almond milk | 14 | icy menthol | 5 |
| cinnamon toast crunch | 14 | custard cream | 5 |
| dark chocolate | 14 | lime juice | 4 |
| cherry ice | 13 | strawberry jelly donut | 4 |
| juicy strawberry | 12 | raspberry jelly | 4 |
| banana pudding | 12 | tropical pineapple | 4 |
| ice tea | 11 | french vanilla coffee | 3 |
| gummy worm | 11 | sweet cream | 3 |
| ice mint | 11 | sweet kiwi | 3 |
| chai tea | 11 | gummy peach | 3 |
| banana strawberry | 10 | strawberry vanilla | 3 |
| blue razz ice | 10 | sweet pineapple | 2 |
| honey mustard | 9 | strawberry cream donut | 2 |
| strawberry jam | 9 | pomegranate ice | 2 |
| cereal milk | 8 | strawberry cookie | 2 |
| cinnamon apple | 8 | sweet cherries | 2 |
| pineapple cake | 7 | pink raspberry | 2 |
| tropical punch | 7 | sweet berry | 2 |
| tropical gummy | 7 | raspberry cherry | 1 |
| apple berry | 7 | pina colada ice | 1 |
| cornbread | 7 | tropical papaya | 1 |
| sweet citrus | 6 | berry peach | 1 |
| iced peach | 6 | blue pear ice | 1 |
| chocolate ice cream | 6 | cola slush | 1 |
| apple cinnamon | 5 | cherry pear | 1 |
| cherry lemon | 5 | sweet peach tea | 1 |
| green apple ice | 5 | icy strawberry | 1 |
| peach pink | 5 | hot cinnamon | 1 |
| white chocolate mocha | 5 | berry medley | 1 |
| strawberry banana ice | 5 | vanilla blood orange | 1 |
| thai tea | 5 | salted caramel donut | 1 |
| fruity menthol | 5 | cherry berry | 1 |
| peach green tea | 5 | juicy melon | 1 |
| sour watermelon | 4 | coconut cream | 1 |
| kiwi berry ice | 4 | sweet vanilla | 1 |
| key lime cookie | 4 | ice peach | 1 |
| blueberry banana | 4 | blue banana | 1 |
| ice cream cookie | 4 | iced green tea | 1 |
| cherry vanilla | 4 | tropical ice | 1 |
| strawberry cake | 3 | strawberry lime cider | 1 |
| redbull ice | 3 | pineapple lime | 1 |
| strawberry guava ice | 3 | sweet tropical | 1 |
| sweet lychee | 3 | cherry strawberry | 1 |
| pineapple coconut | 3 | strawberry whip | 1 |
| watermelon cherry | 3 | pineapple grapefruit ice | 1 |
| sweet raspberry cream | 3 | watermelon peach | 1 |
| peach cream | 3 | sour citrus | 1 |
| butter pecan pie | 3 | juicy papaya | 1 |
| red cherry | 3 | sweet lemonade | 1 |
| kiwi pomegranate | 3 | purple slush ice | 1 |
| kiwi berry | 3 | melon medley | 1 |
| blue lemonade | 3 | creamy marshmallow cereal | 1 |
| juicy berries | 3 | rainbow chew | 1 |
| matcha green tea | 3 | sweet chocolate | 1 |
| sweet watermelon | 3 | rainbow cotton candy | 1 |
| tropical banana | 3 | fruit lemonade | 1 |
| gummy bear ice | 2 | sweet marshmallow | 1 |
| taro milk tea | 2 | tropical watermelon | 1 |
| sour berry | 2 | creamy caramel | 1 |
| apple pear | 2 | sweet cola | 1 |
| peach ice tea | 2 | red fuji apple | 1 |
| sweet apple | 2 | tropical melon | 1 |
| strawberry caramel | 2 | rainbow milkshake | 1 |
| glacier cherry | 2 | peach raspberry | 1 |
| orange limeade | 2 | sweet tangerine | 1 |
| pineapple lemonade | 2 | berry lemonade ice | 1 |
| banana yogurt | 2 | purple slushie ice | 1 |
| ice watermelon limeade | 2 | apple peach ice | 1 |
| peach mint | 2 | red berry | 1 |
| sour peach | 2 | juicy pear | 1 |
| banana watermelon | 2 | honey bear | 1 |
| chocolate donut | 2 | vanilla cookie | 1 |
| gummy cola | 2 | juicy lemonade | 1 |
| sweet orange | 2 | sour lime | 1 |
| strawberry kiwi ice | 2 | juicy kiwi | 1 |
| pink strawberry banana | 2 | caramel donut | 1 |
| peach watermelon | 2 | lime sour | 1 |
| kiwi guava | 2 | sweet cinnamon custard vape | 1 |
| cola slushie | 1 | sweet milk chocolate | 1 |
| butterscotch peach | 1 | icy green apple | 1 |
| peach cherry guava | 1 | strawberry banana donut | 1 |
| ice berry lemonade | 1 | sweet cinnamon | 1 |
| tropical citrus | 1 | fruity cotton candy | 1 |
| raspberry kiwi | 1 | juicy guava | 1 |
| ice cola | 1 |  |  |
| apple cider donut | 1 |  |  |
| yakult yogurt | 1 |  |  |
| peach green tea lemonade | 1 |  |  |
| iced blue raspberry | 1 |  |  |
| vanilla medley | 1 |  |  |
| guava cheesecake mint | 1 |  |  |
| cherry peach lemonade | 1 |  |  |
| strawberry donut | 1 |  |  |
| dragonfruit raspberry iced tea menthol | 1 |  |  |
| peanut butter jelly | 1 |  |  |
| red cola | 1 |  |  |
| peach shark | 1 |  |  |
| cinnamon donut | 1 |  |  |
| tropical red bull | 1 |  |  |
| cranberry raspberry | 1 |  |  |
| cherry lime soda | 1 |  |  |
| cherry starburst | 1 |  |  |
| strawberry muffin | 1 |  |  |
| tropical berry | 1 |  |  |
| cherry cinnamon | 1 |  |  |
| vanilla sugar | 1 |  |  |
| melon berry | 1 |  |  |
| raspberry cream sundae | 1 |  |  |
| mango acai | 1 |  |  |
| butter pudding | 1 |  |  |
| pomegranate berry | 1 |  |  |
| banana vanilla | 1 |  |  |
| vanilla custard donut | 1 |  |  |
| peach pineapple lemonade | 1 |  |  |
| lush ice berry | 1 |  |  |
| peach milkshake | 1 |  |  |
| pink lemonade ice | 1 |  |  |
| rainbow cream | 1 |  |  |
| watermelon raspberry | 1 |  |  |
| banana ice pop | 1 |  |  |
| icy peach | 1 |  |  |
| apple honeydew kiwi | 1 |  |  |
| pink ice guava | 1 |  |  |
| banana kiwi | 1 |  |  |
| vanilla strawberry cake | 1 |  |  |
| juicy cherry | 1 |  |  |
| mango peach guava ice | 1 |  |  |
| pineapple peach lemonade ice | 1 |  |  |
| jelly cola | 1 |  |  |
| cherry pomegranate ice | 1 |  |  |
| brown sugar boba ice | 1 |  |  |
| strawberry orange | 1 |  |  |
| berry banana | 1 |  |  |
| iced kiwi lemon | 1 |  |  |
| butter maple | 1 |  |  |
| tropical pineapple with papaya | 1 |  |  |
| sour worms | 1 |  |  |
| bubblegum raspberry | 1 |  |  |
| ice strawberry mint | 1 |  |  |
| razz ice | 1 |  |  |
| pineapple orange guava | 1 |  |  |
| sweet strawberries | 1 |  |  |
| peach malibu | 1 |  |  |
| cherry bear | 1 |  |  |
| sweet & sour sugar peach | 1 |  |  |
| blue raspberry lemonade menthol | 1 |  |  |
| kiwi pomegranate | 1 |  |  |
| raspberry red licorice | 1 |  |  |
| icy cool melon | 1 |  |  |
| icy honey dew | 1 |  |  |
| chocolate bacon | 1 |  |  |

**Table S3.** New e-cigarette flavors mentioned in both commercial and non-commercial tweets.

| **Intersection** |
| --- |
| banana custard |
| banana ice |
| banana ice cream |
| blue raspberry ice |
| bourbon vanilla |
| butter pecan toffee |
| cherry lime |
| cherry pineapple |
| cola |
| cola ice |
| creamy chocolate |
| fruit medley |
| fruit punch |
| ice cream cake |
| iced coffee |
| juicy watermelon |
| lemonade ice |
| mango ice |
| melon berries |
| mixed berries |
| peach ice |
| peanut butter banana |
| pink colada |
| raspberry pear |
| red apple |
| red berries |
| salted caramel |
| sour apple ice |
| sour cherry |
| strawberry banana |
| strawberry cheesecake |
| strawberry ice cream |
| sugar rush |
| sweet cherry |
| sweet guava |
| sweet peach |
| sweet raspberry |
| sweet tea |
| tropical paradise |
| vanilla cake |
| vanilla ice cream |
| watermelon sugar |

**Figure S1.** Flowchart of new e-cigarette brand identification.

Data Collection and Preprocessing

Split the collected Twitter data into the reference dataset and target dataset

Construct the reference single-word list from the reference dataset

Construct the target single-word lists (commercial and non-commercial) from target datasets

From each target single-word list, remove words that have appeared in the reference single-word list or our previously identified brand list

Manually search each remaining word token on Google to verify if it is a brand

**Figure S2.** Flowchart of e-cigarette flavor identification.

Construct a flavor keyword list based on our previous identified flavor list

Store all tweets containing the phrase "new flavor" into a candidate list

Break the rest of tweets into segments by punctuations and the keyword "and"

Keep only the tweet segments that contain at least one flavor keyword

Remove all segments containing flavors that exist in our previous identified flavor list

Manually check each segment left and record the newly identified flavors
